# Medical rationing choices of laypeople and clinicians are often illogical and inconsistent with their own stated preferences

**DOI:** 10.1371/journal.pone.0322242

**Published:** 2025-05-27

**Authors:** Pedram Heydari, Michelle N. Meyer, Christopher F. Chabris

**Affiliations:** 1 Department of Economics, Northeastern University, Boston, Massachusetts, United States of America; 2 Department of Bioethics and Decision Sciences, Geisinger Health System, Danville, Pennsylvania, United States of America; Lehigh University, UNITED STATES OF AMERICA

## Abstract

The sudden onset, rapid spread, and later surges of the Covid-19 pandemic resulted in shortages of ventilators, pharmaceuticals, and other critical resources, leaving individual clinicians to make rationing decisions for which they had little expertise or training. In two pre-registered experiments with large samples of laypeople (N = 2007) and clinicians (N = 1256), conducted during the first year of the pandemic, we found evidence of two inconsistencies in hypothetical rationing decisions: (1) The choice of which of two patients should receive a medical treatment can be systematically affected by adding a third patient who logically should not receive the treatment (an instance of the attraction effect); (2) Decisions as to which patient should receive the treatment are inconsistent with general rationing policies that participants themselves endorse. We argue that our results provide empirical support for the necessity of predetermined policies administered by independent decision-makers to ensure fairness and consistency, as required by law and ethics, in healthcare rationing choices.

## 1 Introduction

When there are more patients who may benefit from medical treatment than there are human or material resources to provide that care, treatment must inevitably be rationed. Rationing of potentially life-saving resources such as intensive care unit (ICU) beds is not new [1]. However, the sudden onset, rapid spread, and later surges of the Covid-19 pandemic disrupted the global economy, creating shortages in many industries including healthcare. This shortage resulted around the world in periodic rationing of several resources for both Covid-19 and other patients, including ICU beds, ventilators, extracorporeal membrane oxygenation (ECMO) machines, pharmaceuticals, and staff who perform surgeries and other critical procedures—often without formal guidance [2]. For example, in February 2020, frontline doctors in Northern Italy reported substantial distress from having had to make life and death, bedside decisions regarding who would and would not receive care [3,4]. To protect frontline clinicians from the moral and potentially legal responsibility for rationing [5,6], and to ensure consistent, equitable decisions [7], both states and institutions quickly developed algorithms for allocating scarce healthcare resources, in most cases to be implemented by triage officers or committees with no clinical responsibilities for individual patients. By May 2020, more than half of U.S. states had publicly available guidelines for allocating ventilators, although criteria differed substantially [8]. States that were hit hardest by the pandemic eventually invoked those standards, sometimes extending rationing to non-Covid-19 patients [9]. Yet emerging evidence from the second year of the pandemic, when crisis standards of care were most commonly activated [10], suggests that even clinicians who practiced in states or institutions with formal guidelines ultimately made “in-the-moment” rationing decisions on their own, largely detached from those guidelines, and that bypassing frontline clinicians in resource allocation may be “unworkable” [11]. It is therefore important to better understand individual clinician decision-making in this context.

Research in psychology and economics has consistently demonstrated that when people are confronted with specific decisions– such as which consumer product to purchase or which medical treatment to prescribe (in case of doctors)– they can make choices across cases that may be inconsistent with one another [12–15], with normative decision rules, or with a general policy that they endorse [16]. Experiments have shown that choices can be influenced by factors that are not logically relevant, as well as by anxiety and other short-term mental states [17]. Thus, in-the-moment rationing decisions made by clinicians might be subject to the same biases, in which case Covid-19 treatment allocations may not be optimal and might not align with social preferences and policy goals—or even decision-makers’ goals.

In a set of two preregistered studies, one with members of the general public (2007 U.S. crowd workers across two online platforms) and the other with professional clinicians (1256 doctors, other prescribing clinicians, and nurses employed by a large U.S. health system), we explored whether and how decisions about Covid-19 treatment rationing might be subject to context effects, how often a person’s decisions reflected their own preferred rationing policy, and whether making such choices (even in our hypothetical conditions) caused deciders to report stress or any other discomfort.

We hypothesized that the choice of which of multiple Covid-19 patients should receive the only ventilator or drug dose (remdesivir) available will be influenced by one or both of two well-studied context effects on multi-attribute choice: the attraction effect [13], pre-registered as a confirmatory hypothesis, and the compromise effect [14], pre-registered as an exploratory hypothesis. The attraction effect occurs when adding a dominated option D to a set of two options A and B “attracts” choosers to the option that dominates D (say, B), and thus increases their likelihood of choosing B. The compromise effect occurs when the addition of an option E that makes a previous option B now a “compromise” between the new option and the other previous option A increases the choice likelihood of the compromise option (B) relative to option A.

We also evaluated participants’ preferences over treatment rationing policies in two ways: first, we asked them to rate 10 different rationing policies and choose their favorite. Second, we asked if 12 specific factors should be considered in rationing decisions. We used participants’ responses to these questions to explore whether their own rationing choices were consistent with their endorsed policies. We also investigated whether participants demonstrated any self-serving biases in their decisions, i.e., favoring (disfavoring) policies or factors that would serve themselves better (worse) as patients.

Previous studies have demonstrated the presence of both the attraction and compromise effects in various domains, including in consumer products [18–22], monetary lotteries [23], political candidates [24], and policies [25]. However, their relevance for medical decision making, particularly in relation to rationing decisions, has not been investigated to the best of our knowledge. Although Redelmeier and Shafir [15] study context effects in medical decision making, our study is novel in three key aspects: 1) The items of choice in [15] are treatments rather than patients; 2) [15] study other context effects, namely the similarity effect and status quo bias, rather than the attraction and compromise effects. That is, our work concerns completely different ways in which decision-making may depart from normative standards; 3) We also directly consider how decisions may conflict with decision rules or preferences that the same participants endorse, which [15] do not measure.

## 2 Materials and methods

**Participants.** Laypeople (n = 2007, restricted to the U.S.) in Study 1 were crowd workers recruited via two online platforms: Amazon Mechanical Turk (MTurk) and Prolific, with each platform constituting half of our total sample size. With each platform, a single Qualtrics link was used that randomly assigned around 500 participants to each of the two rationing experiments (ventilator or remdesivir). Moreover, within each experiment, Qualtrics was set to evenly and randomly distribute participants across the three conditions (AB, ABD, ABE) in that experiment. All of these participants were paid $1. Both experiments were launched and completed on 08/27/2020. The experiments were advertised as surveys asking for participants’ opinions on an issue of current importance.

Clinicians (n=1256) in Study 2 were doctors, nurses, and other healthcare providers who worked for a large health system in the northeastern United States. These clinicians participated in our survey by responding voluntarily to an email that invited them to take the survey with the prospect of winning a $50 Amazon gift card. This experiment was launched on 01/25/2021, and data collection was closed on 02/13/2021. This experiment was the first part of a two-part survey where the second part was completely unrelated to this study. To reduce selection bias as much as possible, we kept the recruitment email for the survey fairly general and advertised it as a brief survey of the opinions of clinicians regarding possible decisions about treating and studying COVID-19. The ambiguous nature of the recruitment email regarding the content of the study (rationing) as well as the monetary incentive for filling out the survey have potentially reduced the bias in recruiting more prosocial people, an attribute which might have correlation with rationing decisions.

Although these studies were exempt from IRB review under the Common Rule and its requirement of informed consent, we disclosed to prospective participants the main details of the study, including its purpose, and what participants would do and be paid. Participants could quit at any time by closing their internet browser window, and those who completed the survey were deemed to have consented.

**Study format.** The experiments with laypeople (ventilator and remdesivir versions) started with a scenario where multiple Covid-19 patients needed the medical treatment, but there was only enough of that treatment available for one patient. Participants were asked to choose the patient whom they thought should receive the medical treatment. They were then asked to explain why they had chosen the patient that they had chosen in the previous question. Participants then proceeded to answer two attention/robustness check questions which were similar to the first question of the survey except that among the two patients that were available in each of those questions, one was dominated by the other. The experiments then proceeded by evaluating participants rationing policy preferences in a set of three questions. Afterwards, participants were given the Covid Anxiety Scale (CAS) questionnaire [26]. Finally, several demographic information of participants (age, sex, educational attainment, income, political ideology, political party, and religion) were collected at the end of the experiments.

The experiment with clinicians started with the same scenario as the ventilator version of the experiment with the laypeople and followed with the same attention checks and policy preference questions. In this experiment, however, clinicians were not given the CAS questionnaire. We also collected clinicians’ sex and age, as well as their medical position, the number of years working in the medical field, field of specialty, and level of familiarity with scientific research methods.

In both the ventilator and remdesivir rationing scenarios, laypeople were presented with an introductory text that describes the corresponding medical treatment and its role in treating Covid-19 patients. The text further highlighted the scarcity of these treatments, emphasizing that not every patient requiring them may be able to receive them, hence necessitating the implementation of rationing measures. In the introduction of the ventilator scenario presented to health care workers (they did not see a remdesivir scenario), participants were specifically instructed to base their answers on their own personal opinions rather than the policies of their own institution or any other institution.

In both the lay and health care worker experiments, participants were first presented with a situation in which multiple Covid-19 patients need the same scarce medical treatment. Participants were told that based on the patients’ medical records, a computer algorithm had evaluated them in the following two attributes: (1) the likelihood of surviving upon receiving the treatment (expressed as a percentage) and (2) the anticipated years they would be expected to live if they survive Covid-19 (life expectancy). Participants were then asked to decide which patient should receive the only available ventilator (or dose of remdesivir). Note that computer algorithms are frequently used in healthcare and are becoming more prevalent in various tasks (See, for instance, [27]).

To ensure equalized need among the patients in the ventilator scenario, we explicitly stated that each patient would definitely perish if they did not receive a ventilator. In the remdesivir scenario, we sought to achieve a similar balance by stating that all patients met the criteria for the drug as per the government’s decision to approve it for emergency use in treating Covid-19. Furthermore, it was emphasized that all patients were expected to experience a better outcome if they receive remdesivir compared to not receiving the drug.

**Fig 1:**
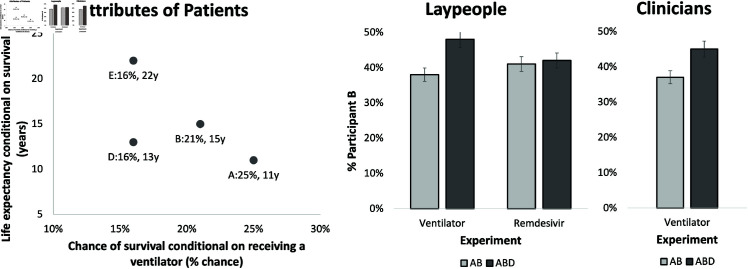
Panel A: Attributes of patients in the ventilator rationing experiments (the patients in the remdesivir experiment were one unit higher in every attribute). Demonstration of the attraction effect in each experiment for laypeople (Panel B) and clinicians (Panel C)–Patient B is chosen more frequently in the ABD condition than in the AB condition.

Both experiments employed a *between-subjects* design in which each participant was randomly assigned to one of three conditions: AB, ABD, and ABE, each representing the set of two or three patients in need of the medical treatment. In the experiment with clinicians, we dropped condition ABE and only used conditions AB and ABD. Patients in the ventilator scenario are described in Table 1 and demonstrated in the graph on the left panel of Figure 1. For the remdesivir scenario, we increased the value of each attribute of these patients by one unit in order to evaluate the robustness of our results against small changes in the attribute values.

**Table 1:** The list of patients in the ventilator experiment. Chance of Survival is conditional on receiving the ventilator. Life Expectancy is conditional on surviving Covid-19

Patient	Chance of Survival (% chance)	Life Expectancy (years)
A	25%	11
B	21%	15
D	16%	13
E	16%	22

We used R to do all statistical analyses. All survey materials, replication data, code, and preregisterations are available on OSF via the following link: https://osf.io/dgv3z/?view_only=81975a0062f145be9b1713b325ed786b+.

## 3 Results

Table 2 displays the percentage of participants who chose each patient, along with the sample size for each condition, across both scenarios.

**Table 2:** Choice shares of patients in different conditions and experiments. Each label in the Condition column refers to the labels of the patients that were presented in that condition. For example, in condition AB, patients A and B were presented.

Experiment	Condition	Patient A	Patient B	Patient D	Patient E	N
Ventilator (Laypeople)	AB	63%	39%	NA	NA	337
	ABD	49%	48%	3%	NA	339
	ABE	55%	29%	NA	26%	332
Remdesivir (Laypeople)	AB	59%	41%	29%	NA	332
	ABD	54%	42%	4%	NA	333
	ABE	46%	29%	NA	25%	334
Ventilator (Clinicians)	AB	63%	37%	NA	NA	618
	ABD	54%	45%	1%	NA	638


As anticipated, choices made by laypeople revealed an attraction effect. Specifically, the choice share for Patient B increased from 39% to 45% in moving from the AB condition to the ABD condition ($\chi^{2}=4.60,df=1,p=\mathrm{.032}$). This overall effect was driven by the ventilator rationing experiment, in which the choice share of Patient B increased from 38% to 48% when moving from the AB condition to the ABD condition ($\chi^{2}=2=7.48,df=1,p=\mathrm{.006}$). We did not observe an attraction effect in the remdesivir rationing experiment (χ2=0.041,df=1,p=.83).

In the experiment with clinicians, we also found support for our confirmatory hypothesis concerning the attraction effect; the choice share of Patient B increased from 37% in the AB condition to 45% in the ABD condition ($\chi^{2}=6.5986,df=1,p=0.010$). See Figure 1 for the visualization of the patients attributes and the choice shares of patient B in different conditions.

To evaluate our exploratory hypothesis, the compromise effect, which was only tested in the laypeople experiment, we compared the choice share of Patient B in condition AB (i.e., %B) with the choice share of Patient B in condition ABE when divided by the sum of the choice shares of Patient A and Patient B in condition ABE (i.e., $\frac{\%B}{\%B+\%A}$). This is equivalent to the comparison of the choice share of Patient B relative to the choice share of Patient A across the two conditions. This hypothesis was not supported in either scenario nor combined (Combined: %B in AB =39%, $\frac{\%B}{\%B+\%A}$ in ABE =37%, $\chi^{2}=0.64,df=1,p=\mathrm{.42}$, Ventilator: %B in AB =37%, $\frac{\%B}{\%B+\%A}$ in ABE =34%, $\chi^{2}=0.36,df=1,p=\mathrm{.55}$, Remdesivir: %B in AB =41%, $\frac{\%B}{\%B+\%A}$ in ABE =39%, $\chi^{2}=0.16,df=1,p=\mathrm{.69}$).

To screen out inattentive participants and/or those who did not find one or both of the attributes that we used to describe patients relevant for their decisions or used them in the opposite order intended (e.g., preferring older patients and inferring patients with higher life expectancies must be older), we included two robustness check questions. The scenario described in these questions was similar to the rationing scenarios outlined above. Specifically, in each question there were two patients who needed the same scarce medical treatment. In robustness check question 1, these patients had equal chances of survival upon receiving the treatment, but one had a higher life expectancy if they survived Covid-19. In robustness check question 2, the two patients had equal life expectancies if they survived, but one of them had a higher chance of survival with the treatment. Participants who selected the dominated patient in either of these questions were either inattentive or did not consider the attribute in which the dominating patient was superior to the dominated patient as relevant to their decision. Consequently, it would be appropriate to exclude these participants from the analysis of this question. In the laypeople and clinicians experiments, 2% and 3% of the participants failed these checks, respectively. After excluding these participants, the statistical significance of our results remained unchanged. However, both the attraction effect and compromise effect slightly intensified in the lay people experiments, while the attraction effect dropped by 1% in the clinicians experiment (for the details of this analysis, see supporting information).

The second part of all our experiments involved posing three questions to our participants regarding policies designed for rationing the corresponding medical treatment in situations akin to those described in the questions where they had to choose a patient directly.

In policy question 1, we presented participants with ten different rationing policies and asked them to rate their degree of approval of each policy on a five-point scale ranging from strongly disapprove to strongly approve, with a neutral midpoint. The table below presents the names of the ten policies as presented to participants in the ventilator rationing experiment (in the remdesivir experiment, the term “ventilator” is replaced by “remdesivir”). It is important to note that participants were only provided with the description of these policies during the experiment, while the names are only included here for clarity. The exact descriptions of these policies can be found in Table 3.

**Table 3:** Policies that participants were asked to rate and pick.

Policy Name	Description
Survival Chance Maximizer	A policy that allocates the ventilator to the patient who has the highest chance of surviving if they receive the ventilator.
Life Expectancy Maximizer	A policy that allocates the ventilator to the patient who has the highest life expectancy if they survive.
Utilitarian	A policy that calculates a score for each patient by multiplying the probability of surviving if the patient receives the ventilator by the patient’s life expectancy if they survive. Then the policy allocates the ventilator to the patient who receives the highest score.
Hybrid	A policy that takes into account the patient’s chance of surviving if they receive the ventilator, the patient’s life expectancy if they survive, and the patient’s current age (giving preference to the youngest patient), and uses a random lottery as a tiebreaker.
Young	A policy that allocates the ventilator to the youngest patient.
Old	A policy that allocates the ventilator to the oldest patient.
Lottery	A policy that allocates the ventilator based on a random lottery that gives each of the patients an equal chance of receiving it.
Neighborhood	A policy that allocates the ventilator to the patient who comes from the most economically disadvantaged neighborhood.
First-Come, First-Served	A policy that allocates the ventilator on a first-come, first-served basis.
Doctor Judgment	A policy that lets the doctors treating the patients use their best judgment to decide which patient will receive the ventilator.

In policy question 2, participants were asked to select which of these (or an “other” policy) they believed a hospital should implement. The Spearman rank correlation between the appropriateness ratings of the policies and the percentage of participants who picked them as their favorite policies is ρ=0.87. Table 4 provides the results of policy questions 1 and 2. Table 12 in the supporting information reports the results of these questions in the laypeople experiment separately for each scenario; ventilator and remdesivir.

**Table 4:** Approval ratings of policies and their choice shares.

Policy Name	% Choosing as Favorite Policy		Mean Approval Rating (SD)	
	Laypeople	Clinicians	Laypeople	Clinicians
Hybrid	23%	24%	3.7 (1.1)	3.4 (0.9)
Survival Chance Maximizer	17%	14%	4.1 (0.8)	3.8 (0.6)
Utilitarian	17%	23%	3.5 (1.1)	3.5 (0.8)
Doctors’ Judgments	17%	24%	3.7 (1.1)	3.6 (0.8)
First-Come, First-Served	9%	2%	3.1 (1.2)	2.6 (1.1)
Life Expectancy Maximizer	5%	3%	3.6 (1.0)	3.7 (0.8)
Lottery	4%	1%	2.8 (1.3)	2.2 (1.1)
Young	3%	1%	3.1 (1.1)	2.9 (1.0)
Neighborhood	1%	0%	2.4 (1.1)	1.7 (0.9)
Old	1%	0%	2.4 (1.0)	2.0 (0.8)
Other	2%	9%	NA	NA

In policy question 3, we presented different pieces of information about Covid-19 patients which could potentially be considered in rationing policies and asked participants to tell us whether in their opinion each piece of information should or should not be considered in rationing situations. Table 5 presents the pieces of information (for the remdesivir experiment, we replaced every instance of ventilator with remdesivir) and their descriptions. Table 6 presents the percentage of participants who indicated each piece of information should be considered in a rationing policy. Note that like the policies, we only gave participants the description of each piece of information. The names used here are chosen for the ease of exposition.

**Table 5:** Pieces of information that were presented to participants.

Information Name	Description
Survival Chance	The patient’s chance of surviving if they receive remdesivir
Life Expectancy	The patient’s life expectancy if they survive Covid-19
Age	The patient’s age
Hospitalization Time	How long the patient has been hospitalized with Covid-19
Habits	Whether the patient’s own previous unhealthy behavior, such as smoking or eating habits, contributed to the severity of their Covid-19 disease
Essential Worker	Whether or not the patient is a health care worker, first responder, or essential worker
Responsibility	How responsible the patient was in following public health guidelines to avoid catching Covid-19
Neighborhood	Whether the patient lives in a poor/economically disadvantaged neighborhood
Race	The patient’s race/ethnicity
Education	The patient’s educational attainment
Income	The patient’s income
Sex	The patient’s sex/gender

**Table 6:** Percentage of participants who have indicated that each piece of information about the patients should be considered in a rationing policy.

Information Name	% of Participants Saying Information Should be Considered	
	Laypeople	Clinicians
Survival Chance	94%	96%
Life Expectancy	82%	91%
Age	73%	77%
Hospitalization Time	65%	57%
Habits	47%	46%
Essential Worker	43%	35%
Responsibility	40%	30%
Neighborhood	15%	4%
Race	6%	2%
Education	6%	4%
Income	5%	1%
Sex	5%	1%

Within this study, some of the policies and pieces of information we included are actually implemented in real-world scenarios for rationing scarce Covid-19 treatments. Specifically, the Hybrid policy is very similar to the Pitt-Penn model hospital policy for rationing scarce medical resources (for details of this policy, see pages 6–9 of the document in the following link: https://www.ccm.pitt.edu/sites/default/files/202305/Allocation_of_Critical_Care_in_Public_Health_Emergency_June2021-FINAL.pdf). Additionally, the neighborhood factor corresponds to one of the primary considerations in the Pittsburgh policy for rationing remdesivir. Therefore, our results on these policies and factors can be potentially important. In fact, the Hybrid policy emerged as the most favored policy. However, the Neighborhood policy, which grants preference to individuals residing in disadvantaged areas, received significant disapproval, and moreover, few of the participants believed that people who come from economically disadvantaged neighborhoods should be treated differently. On the other hand, the First-Come, First-Served policy and the Hospitalization Time factor, which are not used in most real-world policies and are often criticized for their perceived unfairness toward minorities or underserved individuals, were rather popular among our participants.

Rationality demands decisions to be made according to some fixed principles or rationales, which implies that decisions that are made in similar domains should be consistent with one another. As such, in the context of our studies, rationality requires that participants’ chosen patient in the first question of the survey be compatible with their responses to the policy questions. Among the 10 policies above, the Survival Chance Maximizer, Life Expectancy Maximizer, and Utilitarian policies make unambiguous recommendations as to which patient should receive the medical treatment in each of the three experimental conditions we presented (AB, ABD, and ABE). The Survival Chance Maximizer policy recommends Patient A in all three conditions, as this patient has the highest chance of surviving among all patients if they receive the medical treatment. The Life Expectancy Maximizer policy recommends Patient B in the AB and ABD conditions and Patient E in condition ABE, since Patient B and Patient E have the highest life expectancy conditional on surviving Covid-19 in those conditions. The Utilitarian policy recommends Patient B in conditions AB and ABD and Patient E in condition ABE, as these patients achieve the highest score on the product of chance of survival and life expectancy in those conditions.

To assess the prevalence of irrational behavior based on the aforementioned measure of rationality, in our second main analysis we calculated the proportion of participants who chose any of the three policies mentioned above, but whose chosen patient did not align with their chosen policy in their corresponding experimental condition. Overall, among the experiments involving laypeople, 43% of the 770 participants who favored either the Survival Chance Maximizer policy, the Life Expectancy Maximizer policy (which both incorporate only one factor about patients), or the Utilitarian policy as their favorite policy, chose patients that were not consistent with their favorite policy. This percentage slightly increased to 44% (out of 733) after we excluded participants who had chosen a dominated patient in one of the robustness check questions.

In the experiment with clinicians, out of 494 participants whose favorite policies were among the above three policies, 44% chose patients that were inconsistent with their favorite policies. After excluding people who selected a dominated patient in either of the robustness check questions, we ended up with 488 participants who favored one of the mentioned three policies. Even within this group, 43% still selected patients that were inconsistent with their favorite policies. Among these three policies, the Utilitarian policy was both harder to think of and more complex to follow. Therefore, the described inconsistency between the choices of participants who chose this policy might be considered less “irrational.” However, even after focusing only on the 437 participants in the laypeople experiment who chose the Survival Chance Maximizer or Life Expectancy Maximizer policies, we found that 31% had chosen patients who would not have been selected by their preferred rationing policy. This percentage stayed the same after we excluded participants who failed the attention checks. In the experiments with clinicians, focusing on participants who chose one of Survival Chance Maximizer or Life Expectancy Maximizer policies, the percentage dropped to 27% out of 206. This percentage stayed the same after excluding inattentive participants. These percentages are robust to excluding participants who were in the bottom 10th percentile in terms of the time they took to finish the experiment.

To test the robustness of these results even further, we redid the consistency analysis by excluding participants who either chose a dominated patient in the robustness check questions or whose selected policies were not among their highest rated policies, as measured in the policy appropriateness question. In the laypeople experiment, we ended up with 1392 participants. In this subsample, 556 chose one of the three relevant policies, 38% of whom chose patients inconsistent with their selected policies. In the experiment with clinicians, the described exclusion resulted in 1044 remaining participants. 459 of these participants chose one of the three relevant policies, out of which 44% chose a patient that was inconsistent with their selected policies.

In the experiments with laypeople, we also explored the relationship between several demographic variables of participants, as well as their scores on the Covid Anxiety Scale (CAS) [26] in relation to their patient choices and policy preferences. The detailed results of these analyses are presented in supporting information. However, of particular relevance to the aims of our studies is the impact of demographic variables that directly affect how participants would be treated under our rationing policies. For example, younger people would generally benefit from the Young policy while older people would be at a disadvantage. A similar story applies to the Hybrid policy due to the preference it gives to younger people. Therefore, a higher support for these policies among younger people can be interpreted as a self-serving bias, which goes against the idea of impartiality, which is a quality that fair rationing policies should satisfy. Consistent with this bias, in a regression of policy approval ratings on the demographics of participants, we found a significant negative relationship between age of the participants and their ratings of the Hybrid policy (estimated coefficient −0.01,p=0.001) and Young policy (estimated coefficient =−0.01,p<0.001). Also, in a logistic regression of whether or not participants thought each piece of information about Covid-19 patients should be considered in a rationing policy on the demographics of participants, we found a significant negative relationship between the age of the participants and whether they thought a patient’s age should be considered in a rationing policy in policy question 3 (estimated coefficient =−0.0151,p<0.001).

Given the correlation between life expectancy of a patient and their age and the positive bias of older participants towards older patients, it is plausible that older participants might be more likely to approve using life expectancy in a policy or favor the life expectancy maximizer policy. We examined these relationships. The estimated effect of age on the probability of approving life expectancy as a piece of information in a policy is -0.0118 and statistically significant (p = 0.020; see page 7 in the appendix). However, the relationship between age and approval of the life expectancy maximizer as a policy itself is negative but not statistically significant (p = 0.296).

See supporting information for the details of robustness check and demographic analyses.

## 4 Discussion

In two preregistered experiments with the participation of over 3000 laypeople and clinicians, we investigated the prevalence of two well-known choice anomalies–the attraction effect and the compromise effect—in medical rationing decisions. Additionally, we explored whether participants’ rationing decisions were aligned with their own preferred rationing policies.

In both lay and professional respondents, we found evidence for the attraction effect in ventilator allocation. This is significant, since the addition of a patient who, compared to another patient, is a less appropriate recipient in every described attribute (chance of survival conditional on receiving the ventilator, and life expectancy conditional on survival of Covid-19), and is thus an “irrelevant” alternative, nevertheless affects who among the remaining recipients will receive the ventilator. The replication of the attraction effect in the ventilator domain across two different populations (laypeople and clinicians) points to the robustness of the attraction effect in this domain. Given the importance of ventilators in treating a wide variety of clinical conditions in addition to Covid-19, this result has potential practical relevance.

However, we did not find evidence for the attraction effect in the remdesivir scenario with laypeople (and we did not test it with the clinicians). Moreover, we did not find support for the compromise effect in either of the ventilator or remdesivir scenarios among laypeople, the population in which the effect was tested.

Given the similarity of the ventilator and remdesivir scenarios, it is puzzling that the attraction effect was observed in the former but not in the latter scenario. In what follows, we present a few arguments that might explain this contrast. First, despite their similarity, there was an important difference between the two scenarios. Participants in the ventilator scenario were told that patients not served with the treatment would certainly die, but this explanation was absent in the remdesivir scenario. The higher stakes in the ventilator scenario are likely to elicit stronger emotional and cognitive responses, including a greater reliance on dominance as a justification for choice. This, in turn, may amplify the observed attraction effect. In support of this interpretation, [14] demonstrated that requiring participants to justify their choices to others increases the strength of the attraction effect. Similarly, [28] show that binding or incentivized choices, which involve higher stakes, tend to enhance this effect. Together, these findings suggest that more consequential decisions, like those in the ventilator scenario, may produce a stronger attraction effect. Nonetheless, we acknowledge that this explanation remains speculative and should be explored with more care in future research. Second, the absence of a significant effect in the remdesivir scenario may be partially attributed to noisier data and lower statistical power. Less consequential decisions, as in the remdesivir scenario, may reduce participants’ engagement or attention, increasing variability in responses. Additionally, there are indications that the attraction effect was observed in the Prolific portion of the sample but diminished when combined with the MTurk sample, which is known for higher variability and more inattentive responding. Specifically, we found a statistically significant attraction effect in the remdesivir scenario among the participants who were recruited via Prolific (half of our sample) after the exclusion of people who failed the attention checks. In particular, 54 out of 154 participants (35%) in the AB condition and 72 out of 148 participants (49%) in the ABD condition chose Patient B, giving an attraction effect of 14% ($\chi^{2}=5.1822,df=1,p=0.023$). This effect was offset by the absence of an attraction effect among the MTurk participants.

It is important to emphasize that the attraction effect observed in the ventilator scenario was both preregistered and replicated across distinct cohorts, including lay participants and medical practitioners. This replication provides strong evidence for the reliability of the findings in this domain, despite the overall null results observed in the remdesivir scenario.

Analyzing the policy preferences of participants, we found a substantial degree of inconsistency between how participants allocate treatments to the patients before them versus the rationing policy that they endorse the most. Specifically, of the participants whose preferred rationing policies unambiguously recommend an appropriate patient, more than 40% in both experiments choose different patients.

Random errors and deviations from true preferences in one or both of the patient choice and the policy choice questions can lead to inconsistent answers across the two questions. However, such errors are unlikely to be a significant source of inconsistency in our data, since very few participants made an error in the attention check questions (i.e., chose a dominated patient), questions that were very similar to the main patient choice question. Also, our results remain relatively robust after excluding participants who did not pass the attention checks, those who finished the experiment relatively faster, and those who did not select a policy that was one of their highest rated policies. Moreover, given the similarity of the degree of inconsistency in both experiments, it is unlikely that our results are due to sheer random errors. Even if such errors are partially responsible for the observed inconsistency in our data, they must be avoided in actual rationing decisions. One way to avoid such errors is following predetermined rationing policies.

Another explanation for the described inconsistency, which we believe is more likely, is that when deciding which patient should receive the treatment, participants do not necessarily summon pre-defined preferences or policies to make a decision. Rather, they construct their preferences in the given context based on the available options (patients). This idea is consistent with the literature on context-dependent preferences ([29]). Now, when a participant’s revealed preference (choice) in the patient choice question is a function of the available patients, it may conflict with a universal (context-free) policy that they get to endorse later in the experiment.

This explanation suggests that if participants were able to think about their favorite policy prior to the patient choice question, their inconsistency would be reduced. Exploring this idea can be an interesting avenue for future research.

This finding may also be a manifestation of the documented gap in people’s behavior when they are in “hot” versus “cold” mental states (see [30] for a review). In particular, when people judge policies that are to be applied to unidentified people in the future, they may operate in a “cold” and “rational” frame of mind. However, when confronted with the more emotionally charged decision of deciding which individual patient should receive a treatment, they may enter into a “hot” frame in which they summon a different set of rationalizations and heuristics, hence the inconsistent behavior. This result complements the result of [31] who find a discrepancy between the rate with which their participants ration medical treatments at the bedside and policy levels in hypothetical rationing scenarios.This result may also be framed in terms of the documented discrepancy between the way doctors recommend treatment to individual patients versus groups of patients [32,33] as well as the discrepancy between people’s inferred utility functions and their rationing decisions [34]. Similar to our work in spirit, [35] also compared stated principles and stated preferences in a related context, and [36] considered the stability of these principles and choices in the context of Covid-19.

In the design of our studies, the rationing policy questions always came after the choices. This could plausibly result in post hoc rationalization. However, we believe this potential bias does not undermine our findings; rather, it may lead to an underestimation of inconsistencies, which further supports a central point of our paper, that decisions are, if anything, even more inconsistent with preferred policies than our results show. Additionally, reversing the order of the questions would not necessarily eliminate this issue. Participants might then deliberately or unconsciously tend to align their choices with their previously stated policy preferences, thus creating a different but comparable form of consistency bias.

One may, however, object that treating a patient choice that deviates from the favored policy of a participants as an instance of inconsistency might be too harsh. In other words, choosing a policy as as the most favorite policy doesn’t mean not valuing other policies at all. In such a case, one might argue that if a participant’s most favored policy says they should choose a certain patient (A), but all the other arguments/policies she values point towards choosing another patient (B), then it might seem unfair to say the participant was inconsistent in choosing patient B. While we understand this concern, we believe it is essential to clarify the role and definition of a policy within the context of our studies. By definition, a policy is intended to provide a consistent rule to be applied across all cases. As such, selecting a policy as the most important implies a commitment to follow it universally, rather than combining it with other considerations on a case-by-case basis. To account for participants who do not wish to adhere strictly to a single policy, we provided the “Other” option, allowing them to describe a more nuanced approach. In both experiments, a very small percentage of participants chose the “Other” option (2% of laypeople and 9% of clinicians). Moreover, in the policy question, we told participants to assume the policy they choose would be used in a situation like the one that had been described to them earlier in the patient choice question. This suggests that there is little reason for participants to believe that the scenario described in the patient choice question was unusual compared to the general use cases of their favorite policies. Thus, we believe it’s reasonable to expect a “consistent” or rational participant to use their favorite policy to allocate the treatment to the patients presented to them.

The influence of irrelevant alternatives in allocating treatments to patients, as reflected in the attraction effect, combined with the inconsistency of such decisions with the desired policies of our participants, suggest that rationing decisions may be difficult for people (including clinicians) to make on a case by case basis. This is supported by informal examination of participants’ free response comments, which included several reports of feeling anxiety and other negative emotions while making choices in our hypothetical and quite abstract scenarios. For example:

“It was a little bit depressing having to consider these things ...”

“Stressful.”

“I was uncomfortable with playing God.”

“It was disturbing to think about it.”

Being required to apply a pre-established, generally accepted policy in such situations, rather than make decisions involving difficult tradeoffs on the spot, might be expected to reduce such negative emotions.

As a final note, it should be noted that a large fraction of our participants in the clinician experiment were nurses. However, nurses are often integral members of multidisciplinary healthcare teams involved in the development and implementation of crisis standards of care, including COVID-19 rationing policies. For example, [37] and [38] discuss the participation of nurses in these teams, emphasizing their contributions to triage decisions and the ethical frameworks guiding resource allocation. Furthermore, some published guidelines explicitly highlight the need for multidisciplinary teams that include nurse leaders in making these decisions, while others assign this responsibility to senior physicians [5]. Even in cases where nurses are not directly involved in creating guidelines, they frequently manage life-sustaining technologies and play a critical role in implementing triage decisions, including ventilator withdrawal and reallocation [5]. Beyond their involvement in explicit rationing decisions, nurses engage in implicit bedside rationing of their care, which is often referred to as “missed care” or “unfinished care.” This type of rationing involves allocating their time and attention across multiple patients and prioritizing care tasks, often without the guidance of formal frameworks. Evidence from systematic reviews [39] and recent studies, e.g., [40] and [41], indicates that such rationing can have significant consequences for patient safety and quality of care. These findings underscore the relevance of nurses to real-world rationing scenarios, even if their decisions are more implicit than those of physicians managing ventilators or medication distribution.

Although Covid-19 crisis standards of care are not currently active, future developments in Covid-19, such as vaccination and treatment-resistant variants, as well as future pandemics, such as a pandemic influenza, could make them necessary once again. Moreover, health systems that allocate care on the basis of need rather than ability to pay engage in more or less constant rationing [42] and a growing literature similarly documents that implicit rationing routinely, if less visibly, occurs when human resources, such as nurses, are in short supply (e.g., [43]). Good decision-making regarding allocation of scarce health resources therefore remains necessary. Our findings highlight the necessity of designing and communicating treatment allocation policies and implementation of practices that take into account how guidelines are applied (or fail to be applied) by frontline clinicians.

## 5 Results after exclusions

Table 7 presents the choice shares of different patients as well as the sample size in each condition for the laypeople and clincian experiments after excluding the participants who failed the attention/robustness check question (i.e., chose a dominated patient in one of those questions). After these exclusions the attraction effect grew a bit larger in both domains (Ventilator: Patient B in AB=37%, Patient B in $ABD=49\%,\chi^{2}=9.49,df=1,p=\mathrm{.002}$ and Remdesivir: Patient B in AB=41%, Patient B in ABD=45%,χ2=0.88,df=1,p=.35). We continued to fail to observe the compromise effect, although the negative compromise effect shrank (Ventilator: $\chi^{2}=0.15,df=1,p=\mathrm{.70}$, Remdesivir: $\chi^{2}=0.00,df=1,p=1$). With both domains combined, we continued to observe a significant attraction effect (Patient B in AB=39%, Patient B in $ABD=47\%,\chi^{2}=8.54,df=1,p=0.004$) but no compromise effect (χ2=0.15,df =1,p=.70). In the experiment with clinicians, the attractions effect fell by about 1% but remained significant ($\chi^{2}=6.703,df=1,p=0.009$).

**Table 7:** Choice shares of patients in different conditions and experiments conditional on passing the attention checks. Each label in the Condition column refers to the labels of the patients that were presented in that condition. For example, in condition AB, patients A and B were presented

Experiment	Condition	Patient A	Patient B	Patient D	Patient E	N
Ventilator (Laypeople)	AB	63%	37%	NA	NA	319
	ABD	49%	49%	1%	NA	320
	ABE	55%	30%	NA	16%	315
Remdesivir (Laypeople)	AB	59%	41%	29%	NA	306
	ABD	54%	45%	1%	NA	298
	ABE	45%	30%	NA	25%	300
Ventilator (Clinicians)	AB	62%	38%	NA	NA	602
	ABD	55%	45%	0%	NA	620

## 6 Inconsistency analysis after exclusions

**Laypeople experiment.** In the experiment with laypeople, after excluding the 201 people who were in the bottom 10th percentile, we ended up with 685 participants who had chosen one of the three relevant policies, 298 of which (44%) were inconsistent. After also excluding the inattentive participants, 653 of the remaining participants had chosen one of the three relevant policies, out of which 283 (43%) were inconsistent.

**Clinicians experiment.** In the experiment with clinicians, after excluding the 128 people who were in the bottom 10th percentile, we ended up with 437 participants who had chosen one of the three relevant policies, 193 of which (44%) were inconsistent. After also excluding the inattentive participants, 431 of the remaining participants had chosen one of the three relevant policies, out of which 190 (44%) were inconsistent.

## 7 Demographic analysis of the laypeople experiment

Tables 8 and 9 below presents the regression results on the relationship between the approval ratings of policies and the demographics of participants in the experiment with laypeople. Note that participants who have chosen “Prefer not to answer” in any of the demographic questions or have chosen “Other” in the sex, race, or the religion question have been excluded in this analysis (198 people were excluded). Also, people who have chosen multiple races are coded as “Mixed” with the categories being Asian, Black, Hispanic, and White, and people who have chosen a religion are coded as Religious (Religious=1) and non-religious, otherwise (Religious=0). Below are the definition of the other variables in the regression:

**Table 8:** Results of regressing the approval ratings of policies on the demographic variables, part 1.

Policy	Regressor	Estimate	Std. Error	t value	Pr(>|t|)
Hybrid	(Intercept)	3.3008	0.1845	17.89	0.0000
	combined_pol	-0.0844	0.0295	-2.86	0.0043
	CAS_score	0.0004	0.0082	0.04	0.9648
	Age	-0.0075	0.0022	-3.48	0.0005
	SexMale	0.1993	0.0519	3.84	0.0001
	RaceBlack	0.1060	0.1157	0.92	0.3596
	RaceHispanic	-0.0446	0.1300	-0.34	0.7315
	RaceMixed	-0.0789	0.1438	-0.55	0.5834
	RaceWhite	0.0486	0.0812	0.60	0.5491
	Education_years	0.0282	0.0112	2.53	0.0115
	Income_value	0.0021	0.0008	2.62	0.0089
	Religious	-0.0471	0.0561	-0.84	0.4014
Survival Chance Maximizer	(Intercept)	4.0058	0.1418	28.26	0.0000
	combined_pol	-0.0392	0.0227	-1.73	0.0846
	CAS_score	-0.0055	0.0063	-0.87	0.3866
	Age	0.0031	0.0017	1.86	0.0634
	SexMale	0.0468	0.0399	1.17	0.2408
	RaceBlack	0.0975	0.0889	1.10	0.2732
	RaceHispanic	0.1152	0.0999	1.15	0.2490
	RaceMixed1	0.2210	0.1105	2.00	0.0456
	RaceWhite	0.0956	0.0624	1.53	0.1258
	Education_years	-0.0125	0.0086	-1.45	0.1465
	Income_value	0.0015	0.0006	2.33	0.0197
	Religious	0.0160	0.0431	0.37	0.7104
Doctors Judgement	(Intercept)	3.4727	0.1860	18.67	0.0000
	combined_pol	-0.0816	0.0298	-2.74	0.0062
	CAS_score	0.0045	0.0083	0.54	0.5911
	Age	0.0029	0.0022	1.31	0.1911
	SexMale	0.1138	0.0523	2.18	0.0297
	RaceBlack	-0.1261	0.1166	-1.08	0.2799
	RaceHispanic	0.0616	0.1311	0.47	0.6385
	RaceMixed	0.0005	0.1449	0.00	0.9974
	RaceWhite	0.2032	0.0818	2.48	0.0131
	Education_years	-0.0034	0.0112	-0.30	0.7608
	Income_value	-0.0003	0.0008	-0.33	0.7434
	Religious	0.0494	0.0565	0.87	0.3819
Utilitarian	(Intercept)	3.1666	0.1820	17.40	0.0000
	combined_pol	-0.0381	0.0291	-1.31	0.1907
	CAS_score	0.0045	0.0081	0.56	0.5785
	Age	-0.0048	0.0021	-2.27	0.0236
	SexMale	0.0960	0.0512	1.87	0.0612
	RaceBlack	0.1437	0.1142	1.26	0.2085
	RaceHispanic	-0.1069	0.1283	-0.83	0.4047
	RaceMixed	-0.0297	0.1419	-0.21	0.8343
	RaceWhite	0.0328	0.0801	0.41	0.6824
	Education_years	0.0229	0.0110	2.08	0.0378
	Income_value	0.0023	0.0008	2.83	0.0047
	Religious	-0.0283	0.0553	-0.51	0.6097
Life Expectancy Maximizer	(Intercept)	3.5935	0.1763	20.39	0.0000
	combined_pol	0.0383	0.0282	1.36	0.1746
	CAS_score	0.0023	0.0079	0.29	0.7721
	Age	-0.0022	0.0021	-1.04	0.2964
	SexMale	0.0389	0.0496	0.78	0.4332
	RaceBlack	0.2059	0.1106	1.86	0.0627
	RaceHispanic	-0.1587	0.1242	-1.28	0.2016
	RaceMixed	0.0298	0.1374	0.22	0.8282
	RaceWhite	0.0260	0.0776	0.34	0.7375
	Education_years	0.0029	0.0107	0.27	0.7883
	Income_value	-0.0008	0.0008	-1.01	0.3113
	Religious	0.0061	0.0536	0.11	0.9097

**Table 9:** Results of regressing the approval ratings of policies on the demographic variables, part 2.

Policy	Regressor	Estimate	Std. Error	t value	Pr(>|t|)
Young	(Intercept)	3.3017	0.1892	17.45	0.0000
	combined_pol	0.0157	0.0303	0.52	0.6032
	CAS_score	0.0395	0.0084	4.68	0.0000
	Age	-0.0084	0.0022	-3.77	0.0002
	SexMale	0.0104	0.0532	0.19	0.8455
	RaceBlack	0.3842	0.1187	3.24	0.0012
	RaceHispanic	0.2413	0.1334	1.81	0.0706
	RaceMixed	0.0765	0.1475	0.52	0.6039
	RaceWhite	0.1156	0.0833	1.39	0.1651
	Education_years	-0.0119	0.0114	-1.04	0.2981
	Income_value	0.0000	0.0008	0.06	0.9527
	Religious	0.1058	0.0575	1.84	0.0661
Lottery	(Intercept)	3.0430	0.2148	14.17	0.0000
	combined_pol	0.0156	0.0344	0.45	0.6502
	CAS_score	0.0512	0.0096	5.35	0.0000
	Age	-0.0052	0.0025	-2.05	0.0404
	SexMale	-0.1791	0.0605	-2.96	0.0031
	RaceBlack	0.2004	0.1348	1.49	0.1372
	RaceHispanic	0.1404	0.1514	0.93	0.3540
	RaceMixed	0.0557	0.1674	0.33	0.7394
	RaceWhite	0.0320	0.0945	0.34	0.7348
	Education_years	-0.0110	0.0130	-0.85	0.3958
	Income_value	-0.0001	0.0009	-0.11	0.9120
	Religious	0.1155	0.0653	1.77	0.0771
First-Come, First-Served	(Intercept)	3.0906	0.2015	15.34	0.0000
	combined_pol	0.1003	0.0322	3.11	0.0019
	CAS_score	0.0154	0.0090	1.72	0.0861
	Age	0.0035	0.0024	1.47	0.1412
	SexMale	-0.1116	0.0567	-1.97	0.0491
	RaceBlack	0.1820	0.1264	1.44	0.1500
	RaceHispanic	-0.1053	0.1420	-0.74	0.4586
	RaceMixed	-0.0144	0.1570	-0.09	0.9269
	RaceWhite	0.0501	0.0887	0.57	0.5717
	Education_years	-0.0107	0.0122	-0.88	0.3781
	Income_value	0.0004	0.0009	0.40	0.6917
	Religious	0.1198	0.0612	1.96	0.0506
Neighborhood	(Intercept)	2.5960	0.1771	14.66	0.0000
	combined_pol	-0.1983	0.0283	.00	0.0000
	CAS_score	0.0716	0.0079	9.06	0.0000
	Age	-0.0126	0.0021	-6.05	0.0000
	SexMale	0.0394	0.0498	0.79	0.4287
	RaceBlack	0.1483	0.1111	1.34	0.1820
	RaceHispanic	-0.1145	0.1248	-0.92	0.3589
	RaceMixed	-0.0152	0.1380	-0.11	0.9123
	RaceWhite	-0.3026	0.0779	-3.88	0.0001
	Education_years	0.0277	0.0107	2.59	0.0098
	Income_value	-0.0030	0.0008	-3.79	0.0002
	Religious	0.1012	0.0538	1.88	0.0602
Old	(Intercept)	2.6149	0.1691	15.46	0.0000
	combined_pol	0.0585	0.0271	2.16	0.0309
	CAS_score	0.0713	0.0075	9.45	0.0000
	Age	0.0008	0.0020	0.40	0.6904
	SexMale	0.0124	0.0476	0.26	0.7945
	RaceBlack	0.3381	0.1061	3.19	0.0015
	RaceHispanic	0.0196	0.1192	0.16	0.8693
	RaceMixed	-0.0272	0.1318	-0.21	0.8365
	RaceWhite	-0.1177	0.0744	-1.58	0.1141
	Education_years	-0.0211	0.0102	-2.07	0.0390
	Income_value	-0.0018	0.0007	-2.46	0.0140
	Religious	0.2245	0.0514	4.37	0.0000

Combined pol=Political attitude index (a la. [44]). This is calculated by averaging the z-scores of the answers to the political ideology and political party questions. Political ideology is measured on a 5-point scale ranging from Very Liberal (1), Liberal (2), Neutral (3), Conservative (4), Very Conservative (5). Political Party is measured on a 7-point scale ranging from Very Democrat (1), Democrat (2), Somewhat Democrat (3), Neutral (4), Somewhat Conservative (5), Conservative (6), Very Conservative (7).

CAS_score: Covid anxiety scale. This is the z-score of the score on the Covid anxiety scale [26].

Education years: Less than High School=10, High School Degree=12, Some College=14, College=16 Some Graduate=18, Graduate Degree=20

Income value: Less than 20k=10, Between 20k and 40k=30, Between 40k and 60k=50, Between 60k and 80k=70, Between 80k and 100k=90, More than 100k=110

Tables 10 and 11 below presents the logistic regression results on the relationship between the opinions on whether or not a piece of information should be considered and the demographics of participants in the laypeople experiment. Table 12 presents policy preferences of laypeople across the two experimental conditions; ventilator and remdesivir. Finally, the appendix ends with the survey materials, first for the laypeople experiment and then, for the experiment with clinicians.

**Table 10:** Results of logistic regression of if different pieces of information must be used in a rationing policy on the demographic variables, part 1.

Information	Regressor	Estimate	Std. Error	z value	Pr(>|z|)
Income	(Intercept)	-2.6758	0.7727	-3.46	0.0005
	combined_pol	-0.0064	0.1332	-0.05	0.9618
	CAS_score	0.1566	0.0233	6.71	0.0000
	Age	-0.0479	0.0123	-3.89	0.0001
	SexMale	0.4818	0.2293	2.10	0.0356
	RaceBlack	0.2625	0.4043	0.65	0.5161
	RaceHispanic	-0.2098	0.4942	-0.42	0.6711
	RaceMixed	0.2359	0.5097	0.46	0.6435
	RaceWhite	-0.3986	0.3152	-1.26	0.2060
	Education_years	0.0248	0.0498	0.50	0.6190
	Income_value	0.0027	0.0036	0.76	0.4467
	Religious	0.5007	0.2506	2.00	0.0457
Sex	(Intercept)	-3.7122	0.7982	-4.65	0.0000
	combined_pol	0.2923	0.1272	2.30	0.0215
	CAS_score	0.2013	0.0227	8.85	0.0000
	Age	-0.0259	0.0112	-2.32	0.0202
	SexMale	0.5873	0.2346	2.50	0.0123
	RaceBlack	-0.2249	0.4730	-0.48	0.6344
	RaceHispanic	0.2390	0.4866	0.49	0.6233
	RaceMixed	-0.3569	0.6692	-0.53	0.5938
	RaceWhite	-0.2504	0.3357	-0.75	0.4559
	Education_years	0.0040	0.0502	0.08	0.9369
	Income_value	0.0070	0.0037	1.87	0.0616
	Religious	0.6873	0.2651	2.59	0.0095
Race	(Intercept)	-4.5856	0.7816	-5.87	0.0000
	combined_pol	0.2268	0.1261	1.80	0.0720
	CAS_score	0.1758	0.0225	7.82	0.0000
	Age	-0.0384	0.0115	-3.33	0.0009
	SexMale	0.4013	0.2278	1.76	0.0782
	RaceBlack	0.1171	0.4350	0.27	0.7877
	RaceHispanic	-0.2073	0.5349	-0.39	0.6983
	RaceMixed	-0.3846	0.6601	-0.58	0.5601
	RaceWhite	-0.2104	0.3230	-0.65	0.5149
	Education_years	0.1171	0.0481	2.44	0.0149
	Income_value	0.0036	0.0036	1.00	0.3154
	Religious	0.5268	0.2565	2.05	0.0400
Avoid	(Intercept)	0.9199	0.3551	2.59	0.0096
	combined_pol	-0.0445	0.0571	-0.78	0.4356
	CAS_score	0.0569	0.0156	3.66	0.0003
	Age	-0.0173	0.0043	-4.03	0.0001
	SexMale	0.2557	0.0994	2.57	0.0101
	RaceBlack	-0.6123	0.2186	-2.80	0.0051
	RaceHispanic	-0.6530	0.2444	-2.67	0.0075
	RaceMixed	-0.5294	0.2694	-1.97	0.0494
	RaceWhite	-0.6698	0.1525	-4.39	0.0000
	Education_years	-0.0279	0.0216	-1.29	0.1956
	Income_value	0.0006	0.0016	0.36	0.7215
	Religious	0.0806	0.1077	0.75	0.4542
Lifestyle	(Intercept)	0.4294	0.3524	1.22	0.2230
	combined_pol	0.2505	0.0567	4.42	0.0000
	CAS_score	-0.0060	0.0157	-0.39	0.6999
	Age	-0.0169	0.0042	-4.04	0.0001
	SexMale	0.5097	0.0983	5.18	0.0000
	RaceBlack	-0.5085	0.2224	-2.29	0.0222
	RaceHispanic	-0.8930	0.2485	-3.59	0.0003
	RaceMixed	-0.7046	0.2728	-2.58	0.0098
	RaceWhite	-0.8948	0.1593	-5.62	0.0000
	Education_years	0.0399	0.0212	1.88	0.0604
	Income_value	-0.0007	0.0015	-0.48	0.6293
	Religious	0.0084	0.1070	0.08	0.9377
Neighborhood	(Intercept)	-2.1486	0.4749	-4.52	0.0000
	combined_pol	-0.1943	0.0813	-2.39	0.0169
	CAS_score	0.0989	0.0176	5.61	0.0000
	Age	-0.0315	0.0066	-4.77	0.0000
	SexMale	0.0687	0.1365	0.50	0.6149
	RaceBlack	0.1424	0.2676	0.53	0.5947
	RaceHispanic	0.2170	0.2947	0.74	0.4615
	RaceMixed	0.3531	0.3188	1.11	0.2680
	RaceWhite	-0.3245	0.1953	-1.66	0.0966
	Education_years	0.0720	0.0293	2.46	0.0140
	Income_value	0.0016	0.0021	0.74	0.4609
	Religious	0.3646	0.1476	2.47	0.0135

**Table 11:** Results of logistic regression of if different pieces of information must be used in a rationing policy on the demographic variables, part 2.

Information	Regressor	Estimate	Std. Error	z value	Pr(>|z|)
Age	(Intercept)	1.7148	0.3940	4.35	0.0000
	combined_pol	-0.0387	0.0614	-0.63	0.5280
	CAS_score	0.0071	0.0175	0.40	0.6862
	Age	-0.0151	0.0044	-3.40	0.0007
	SexMale	0.1380	0.1097	1.26	0.2087
	RaceBlack	-0.3630	0.2419	-1.50	0.1334
	RaceHispanic	-0.2786	0.2768	-1.01	0.3141
	RaceMixed	-0.0405	0.3161	-0.13	0.8980
	RaceWhite	-0.0804	0.1801	-0.45	0.6552
	Education_years	-0.0123	0.0234	-0.52	0.5997
	Income_value	0.0033	0.0017	1.91	0.0567
	Religious	-0.2050	0.1185	-1.73	0.0835
Life Expectancy	(Intercept)	2.0440	0.4579	4.46	0.0000
	combined_pol	-0.0896	0.0707	-1.27	0.2050
	CAS_score	-0.0414	0.0182	-2.27	0.0232
	Age	-0.0118	0.0051	-2.32	0.0205
	SexMale	0.1807	0.1268	1.43	0.1541
	RaceBlack	-0.5632	0.2846	-1.98	0.0478
	RaceHispanic	-0.3348	0.3304	-1.01	0.3109
	RaceMixed	-0.4328	0.3526	-1.23	0.2197
	RaceWhite	-0.2456	0.2198	-1.12	0.2640
	Education_years	0.0023	0.0270	0.09	0.9322
	Income_value	0.0021	0.0020	1.03	0.3031
	Religious	0.0456	0.1370	0.33	0.7394
Survival Chance	(Intercept)	3.8056	0.7551	5.04	0.0000
	combined_pol	-0.1744	0.1124	-1.55	0.1205
	CAS_score	-0.0736	0.0244	-3.02	0.0026
	Age	-0.0033	0.0084	-0.39	0.6958
	SexMale	-0.2952	0.2041	-1.45	0.1479
	RaceBlack	-1.3293	0.4390	-3.03	0.0025
	RaceHispanic	-0.2163	0.5896	-0.37	0.7137
	RaceMixed	-0.6824	0.5910	-1.15	0.2482
	RaceWhite	-0.3870	0.3926	-0.99	0.3242
	Education_years	-0.0019	0.0437	-0.04	0.9655
	Income_value	0.0025	0.0033	0.77	0.4387
	Religious	-0.4654	0.2313	-2.01	0.0442
Hospitalization Time	(Intercept)	2.1667	0.3684	5.88	0.0000
	combined_pol	0.0677	0.0577	1.17	0.2403
	CAS_score	0.0293	0.0168	1.74	0.0816
	Age	-0.0202	0.0042	-4.79	0.0000
	SexMale	-0.2388	0.1019	-2.34	0.0191
	RaceBlack	-0.0513	0.2336	-0.22	0.8261
	RaceHispanic	-0.3753	0.2549	-1.47	0.1409
	RaceMixed	0.1298	0.3008	0.43	0.6662
	RaceWhite	-0.1526	0.1638	-0.93	0.3514
	Education_years	-0.0419	0.0218	-1.92	0.0546
	Income_value	-0.0002	0.0016	-0.14	0.8921
	Religious	0.1534	0.1102	1.39	0.1639
Essential Worker	(Intercept)	-0.5404	0.3464	-1.56	0.1188
	combined_pol	-0.0929	0.0558	-1.67	0.0955
	CAS_score	0.0453	0.0154	2.93	0.0033
	Age	-0.0016	0.0041	-0.38	0.7028
	SexMale	0.0482	0.0978	0.49	0.6222
	RaceBlack	-0.6802	0.2174	-3.13	0.0018
	RaceHispanic	-0.4800	0.2419	-1.98	0.0472
	RaceMixed	-0.3287	0.2668	-1.23	0.2178
	RaceWhite	-0.7109	0.1522	-4.67	0.0000
	Education_years	0.0407	0.0210	1.94	0.0526
	Income_value	0.0025	0.0015	1.63	0.1033
	Religious	0.0388	0.1055	0.37	0.7133
Education	(Intercept)	-4.1734	0.7591	-5.50	0.0000
	combined_pol	0.3693	0.1231	3.00	0.0027
	CAS_score	0.1868	0.0222	8.40	0.0000
	Age	-0.0353	0.0111	-3.17	0.0015
	SexMale	0.5710	0.2252	2.54	0.0112
	RaceBlack	0.2418	0.4101	0.59	0.5554
	RaceHispanic	-0.6610	0.5693	-1.16	0.2456
	RaceMixed	-0.5342	0.6555	-0.82	0.4151
	RaceWhite	-0.3500	0.3096	-1.13	0.2583
	Education_years	0.0841	0.0474	1.78	0.0759
	Income_value	0.0052	0.0035	1.47	0.1423
	Religious	0.4359	0.2499	1.74	0.0812

**Table 12:** Policy preferences of laypeople across the two experimental conditions; ventilator and remdesivir.

Policy Name	% Choosing as Favorite Policy		Mean Approval Rating (SD)	
	Ventilator	Remdesivir	Ventilator	Remdesivir
Hybrid	23%	24%	3.7 (1.1)	3.7 (1.1)
Survival Chance Maximizer	17%	18%	4.1 (0.8)	4.1 (0.0)
Utilitarian	16%	17%	3.5 (1.1)	3.6 (1.1)
Doctors’ Judgments	18%	16%	3.8 (1.1)	3.7 (1.1)
First-Come, First-Served	11%	8%	3.2 (1.2)	3.0 (1.2)
Life Expectancy Maximizer	4%	5%	3.5 (1.0)	3.6 (1.0)
Lottery	3%	6%	2.7 (1.3)	2.9 (1.3)
Young	4%	3%	3.1 (1.1)	3.1 (1.1)
Neighborhood	1%	1%	2.3 (1.1)	2.5 (1.5)
Old	0%	0%	2.4 (1.0)	2.0 (0.8)
Other	2%	2%	NA	NA

## References

[1] Sinuff T, Kahnamoui K, Cook DJ, Luce JM, Levy MM, Values Ethics and Rationing in Critical Care Task Force. Rationing critical care beds: a systematic review. Crit Care Med. 2004;32(7):1588–97. doi: 10.1097/01.ccm.0000130175.38521.9f 15241106

[2] Cha AE, Bernstein L, Sellers FS, Harris S. Faced with a crush of patients, besieged NYC hospitals struggle with life-or-death decisions. The Washington Post, 31 March 2020, [cited 2023 Oct 17]. https://www.washingtonpost.com/health/2020/03/31/new-york-city-hospitals-coronavirus/

[3] Ore J. COVID-19 forces Italian doctors to make life-and-death choices about rationing care CBC. 13 March, 2020, [cited 2023 Oct 17]. Available from: https://www.cbc.ca/radio/day6/

[4] Ferraresi M. A Coronavirus cautionary tale from Italy: don’t do what we did. Boston Globe. 2020 March 13, [cited 2023 Oct 17]. Available from: https://www.bostonglobe.com/

[5] Truog RD, Mitchell C, Daley GQ. The toughest triage - allocating ventilators in a pandemic. N Engl J Med. 2020;382(21):1973–5. doi: 10.1056/NEJMp2005689 32202721

[6] Hoffman S. Responders’ responsibility: liability and immunity in public health emergencies. Geo LJ. 2007;96:1913.

[7] Schmidt H, Roberts DE, Eneanya ND. Rationing, racism and justice: advancing the debate around “colourblind” COVID-19 ventilator allocation. J Med Ethics. 2022;48(2):126–30. doi: 10.1136/medethics-2020-106856 33408091 PMC7789208

[8] Piscitello GM, Kapania EM, Miller WD, Rojas JC, Siegler M, Parker WF. Variation in ventilator allocation guidelines by US State during the Coronavirus Disease 2019 Pandemic: a systematic review. JAMA Netw Open. 2020;3(6):e2012606. doi: 10.1001/jamanetworkopen.2020.12606 32558916 PMC7305526

[9] Calfas J. Idaho hospitals, overwhelmed with covid-19 patients, may begin rationing care. The Wall Street J. 2021 Sep 16, [cited 2023 Oct 17]. Available from: https://www.wsj.com/

[10] TRACIE. Crisis standards of care during COVID-19 summary of state actions1 March 2022. HHS, Administration for Strategic Preparedness and Response (ASPR). [cited 2023 Oct 17]. Available from: https://files.asprtracie.hhs.gov/

[11] Butler CR, Wightman AG, Taylor JS, Hick JL, O’Hare AM. Experiences of US clinicians contending with health care resource scarcity during the COVID-19 pandemic, December 2020 to December 2021. JAMA Netw Open. 2023;6(6):e2318810. doi: 10.1001/jamanetworkopen.2023.18810 37326986 PMC10276299

[12] Tversky A. Elimination by aspects: a theory of choice. Psychol Rev. 1972;79(4):281–99. doi: 10.1037/h0032955

[13] Huber J, Payne JW, Puto C. Adding asymmetrically dominated alternatives: violations of regularity and the similarity hypothesis. J Consum Res. 1982;9(1):90. doi: 10.1086/208899

[14] Simonson I. Choice based on reasons: the case of attraction and compromise effects. J Consum Res. 1989;16(2):158–74.

[15] Redelmeier DA, Shafir E. Medical decision making in situations that offer multiple alternatives. JAMA. 1995;273(4):302–5. doi: 10.1001/jama.1995.03520280048038 7815657

[16] Nielsen K, Rehbeck J. When choices are mistakes. Am Econ Rev. 2022;112(7):2237–68. doi: 10.1257/aer.20201550

[17] Loewenstein G. Emotions in economic theory and economic behavior. Am Econ Rev. 2000;90(2):426–32. doi: 10.1257/aer.90.2.426

[18] Simonson I, Tversky A. Choice in context: tradeoff contrast and extremeness aversion. J Market Res. 1992;29(3):281. doi: 10.2307/3172740

[19] Ariely D, Wallsten TS. Seeking subjective dominance in multidimensional space: an explanation of the asymmetric dominance effect. Organiz Behav Hum Decis Processes. 1995;63(3):223–32. doi: 10.1006/obhd.1995.1075

[20] Herne K. Testing the reference-dependent model: an experiment on asymmetrically dominated reference points. J Behav Decis Making. 1998;11(3):181–92. doi: 10.1002/(sici)1099-0771(199809)11:3<181::aid-bdm295>3.0.co;2-t

[21] Doyle JR, O’Connor DJ, Reynolds GM, Bottomley PA. The robustness of the asymmetrically dominated effect: buying frames, phantom alternatives, and in-store purchases. Psychol Mark. 1999;16(3):225–43. doi: 10.1002/(sici)1520-6793(199905)16:3<225::aid-mar3>3.0.co;2-x

[22] Sharpe KM, Staelin R, Huber J. Using extremeness aversion to fight obesity: policy implications of context dependent demand. J Consum Res. 2008;35(3):406–22. doi: 10.1086/587631

[23] Herne K. The effects of decoy gambles on individual choice. Exp Econ. 1999;2(1):31–40.

[24] Sue O’Curry YP, Pitts R. The attraction effect and political choice in two elections. J Consum Psychol. 1995;4(1):85–101. doi: 10.1207/s15327663jcp0401_04

[25] Herne K. Decoy alternatives in policy choices: Asymmetric domination and compromise effects. Eur J Politic Econ. 1997;13(3):575–89. doi: 10.1016/s0176-2680(97)00020-7

[26] Lee SA. Coronavirus anxiety scale: a brief mental health screener for COVID-19 related anxiety. Death Stud. 2020;44(7):393–401. doi: 10.1080/07481187.2020.1748481 32299304

[27] Balcı MA, Batrancea LM, Akgüller Ö, Nichita A. A series-based deep learning approach to lung nodule image classification. Cancers (Basel). 2023;15(3):843. doi: 10.3390/cancers15030843 36765801 PMC9913559

[28] Lichters M, Bengart P, Sarstedt M, Vogt B. What really matters in attraction effect research: when choices have economic consequences. Mark Lett. 2015;28(1):127–38. doi: 10.1007/s11002-015-9394-6

[29] Lichtenstein S, Slovic P. The construction of preference: an overview. The construction of preference. Vol. 1. 2006.

[30] Loewenstein G. Hot-cold empathy gaps and medical decision making. Health Psychol. 2005;24(4S):S49-56. doi: 10.1037/0278-6133.24.4.S49 16045419

[31] Persson E, Andersson D, Back L, Davidson T, Johannisson E, Tinghög G. Discrepancy between health care rationing at the bedside and policy level. Med Decis Making. 2018;38(7):881–7. doi: 10.1177/0272989X18793637 30198412

[32] Redelmeier DA, Tversky A. Discrepancy between medical decisions for individual patients and for groups. N Engl J Med. 1990;322(16):1162–4. doi: 10.1056/NEJM199004193221620 2320089

[33] DeKay ML, Nickerson CA, Ubel PA, Hershey JC, Spranca MD, Asch DA. Further explorations of medical decisions for individuals and for groups. Med Decis Making. 2000;20(1):39–44. doi: 10.1177/0272989X0002000105 10638535

[34] Ubel PA, Loewenstein G, Scanlon D, Kamlet M. Individual utilities are inconsistent with rationing choices: a partial explanation of why Oregon’s cost-effectiveness list failed. Med Decis Making. 1996;16(2):108–16. doi: 10.1177/0272989X9601600202 8778528

[35] Arroyos-Calvera D, Covey J, Loomes G, McDonald R. The efficiency-equity trade-off, self-interest, and moral principles in health and safety valuation. Soc Sci Med. 2019;238:112477. doi: 10.1016/j.socscimed.2019.112477 31434030

[36] Arroyos-Calvera D, Covey J, McDonald R. Are distributional preferences for safety stable? A longitudinal analysis before and after the COVID-19 outbreak. Soc Sci Med. 2023;324:115855. doi: 10.1016/j.socscimed.2023.115855 37001277 PMC10035807

[37] Morley G, Grady C, McCarthy J, Ulrich CM. Covid-19: ethical challenges for nurses. Hastings Center Rep. 2020;50(3):35–9. 10.1002/hast.1110 PMC7272859 32410225

[38] Mason DJ. Lessons from COVID-19 about rationing care. JAMA Health Forum. 2020;1(9):e201207. doi: 10.1001/jamahealthforum.2020.1207 36218740

[39] Papastavrou E, Andreou P, Efstathiou G. Rationing of nursing care and nurse-patient outcomes: a systematic review of quantitative studies. Int J Health Plann Manage. 2014;29(1):3–25. doi: 10.1002/hpm.2160 23296644

[40] Jones P, Drummond PD. A summary of current findings on quality of life domains and a proposal for their inclusion in clinical interventions. Front Psychol. 2021;12:747435. doi: 10.3389/fpsyg.2021.747435 34777139 PMC8586497

[41] Scott PA, Harvey C, Felzmann H, Suhonen R, Habermann M, Halvorsen K, et al. Resource allocation and rationing in nursing care: a discussion paper. Nurs Ethics. 2019;26(5):1528–39. 29607703 10.1177/0969733018759831 PMC6681425

[42] Andoh B. Rationing of resources in the National Health Service. Medico-Legal J. 2023. 10.1177/00258172231159756 37288529

[43] Renner A, Ausserhofer D, Zúñiga F, Simon M, Serdaly C, Favez L. Increasing implicit rationing of care in nursing homes: a time-series cross- sectional analysis. Int J Nurs Stud. 2022;134:104320. 35868214 10.1016/j.ijnurstu.2022.104320

[44] Kahan DM, Peters E, Dawson EC, Slovic P. Motivated numeracy and enlightened self-government. Behav Publ Policy. 2017;1(1):54–86.

